# Impact of female adult eating disorder inpatients’ attitudes to compulsive exercise on outcome at discharge and follow-up

**DOI:** 10.1186/s40337-016-0096-0

**Published:** 2016-03-09

**Authors:** Marit Danielsen, Øyvind Rø, Ulla Romild, Sigrid Bjørnelv

**Affiliations:** 1 Department of Neuroscience, Faculty of Medicine, Norwegian University of Science and Technology, 7491 Trondheim, Norway; 2 Eating Disorder Unit, Department of Psychiatry, Levanger Hospital, Hospital Trust Nord-Trøndelag, NO-7600 Levanger, Norway; 3 Regional Department for Eating Disorders, Division of Mental Health and Addiction, Oslo University Hospital, Oslo, Norway; 4 Division of Mental Health and Addiction, Institute of Clinical Medicine, University of Oslo, Levanger, Norway; 5 Department of Research and Development, Levanger Hospital, Hospital Trust Nord-Trøndelag, NO-7600 Levanger, Norway

**Keywords:** Eating disorders, Attitudes, Compulsive exercise, Inpatient, Treatment, Outcome

## Abstract

**Background:**

The link between compulsive exercise and eating disorders is well known, but research with clinical samples has been limited. The purpose of the study was to investigate changes in attitudes towards compulsive exercise and its impact on outcome at follow-up in female adult hospitalised patients with eating disorders.

**Methods:**

The sample consisted of 78 patients: Diagnostic distribution: anorexia nervosa 59 % (*n* = 46), approximately 22 % (*n* = 16) in bulimia nervosa, and Eating Disorder not Otherwise Specified respectively. The average follow-up period was 26 months (*SD* =15 months). Compulsive exercise was measured by the Exercise and Eating Disorder (EED) questionnaire. Other measures were the Eating Disorder Inventory (EDI-2), Body Attitude Test (BAT), Symptom Checklist (SCL-90), Inventory of Interpersonal Problems (IIP 64), Beck Depression Inventory (BDI), and body mass index (BMI). Outcome measures were EDI-2 and BMI (patients with admission BMI ≤ 18.5). Paired sample *t*-tests and mixed model regression analysis were conducted to investigate changes in compulsive exercise and predictors of outcome respectively.

**Results:**

All measures revealed significant improvements (*p* < .01 – *p* < .001) from admission to follow-up. EED scores significantly predicted changes in EDI-2 scores and BMI (*p* < .01 and *p* < .001 respectively). Other significant predictors were BAT, SCL-90, IIP-64, BMI (*p* < .01–.001) (EDI-2 as outcome measure), and BAT and BDI (*p* < .001) (BMI as outcome measure).

**Conclusions:**

The results demonstrated significant improvements in attitudes towards compulsive exercise during treatment and follow-up. The change in compulsive exercise scores predicted the longer-term course of eating disorder symptoms and BMI.

## Background

The DSM-IV and DSM-5 diagnostic criteria highlight excessive or compulsive elements of exercise as recurrent inappropriate behaviour in patients with eating disorders [1, 2]. Such behaviour is characterized as inappropriate when it significantly interferes with a person’s life and persists despite injury or medical complications. In cases of anorexia nervosa (AN), the behaviour is described as a cause of weight loss, and in cases of bulimia nervosa (BN) as compensatory behaviour. The only difference between the two editions is that the frequency of compensating behaviour in DSM-5 is reduced from twice a week to weekly [1, 2]. Frequent and intensive physical activity is common in a number of human groups, such as athletes. However, in patients with eating disorders it has been suggested that negative and compulsive attitudes towards exercise are more closely related to symptoms of eating disorders than the excessive amounts of exercise [3, 4].

Numerous definitions and terms have been used in the literature to describe compulsive and/or excessive exercise, yet inconsistency in the definitions renders it difficult for readers to know whether different studies refer to the same phenomenon [5]. Further, this inconsistency may account for the wide range of prevalence rates reported. Two studies observed an overall prevalence of excessive exercise of 39 and 45 % in transdiagnostic samples, with rates varying according to diagnostic type [6, 7]. In one of the samples, the prevalence was as high as 80 % for patients diagnosed with restrictive anorexia nervosa (AN), 43 % in AN binge-eating/purging subtype, 39. % in bulimia nervosa (BN), and 32 % in eating disorders not otherwise specified (EDNOS) (6). Other studies have shown smaller differences between diagnostic groups [8–10].

There has been an increased interest in the nature of excessive and/or compulsive exercise in eating disorders and the association with symptomatology and treatment outcome [3, 4]. Such attitudes and behaviour have been investigated in treatment programmes with and without exercise interventions. A drive for compulsive exercise present at discharge has been reported as a negative prognostic factor for the long-term course of AN [11]. Carter and colleagues [12] investigated high-level exercise in the first three months after discharge from treatment, and found a significant association with the probability of relapse. Associations between objective measures of amount of exercise (at initiation of treatment) and eating disorder pathology have been investigated. In a review, Gummer and colleagues [13] reported that results were partly conflicting as to whether associations existed. In an inpatient treatment programme (without exercise intervention) the results revealed that a lower amount of pre-treatment exercise was a significant predictor of improvement in eating disorder symptoms [6].

Specific exercise interventions are not commonly incorporated in treatment programmes, and few studies have explored the influence of outcome when such interventions are included [12–14]. In one study with AN, BN, EDNOS, a significant positive correlation was found between reduced exercise dependency and reduced eating disorder symptoms during inpatient treatment [14]. Literature relating to excessive exercise and AN [15, 16] and in transdiagnostic samples [17] has been summarized. A total of 13 studies were evaluated, most of which were characterized by small sample sizes and considerable heterogeneity in definitions, outcome measures, and type of intervention. Despite their methodological heterogeneity, the studies indicate that exercise interventions as a part of treatment have no significant adverse impacts on weight gain for AN patients [15–17]. Four studies found a positive association between exercise interventions and reduced eating disorder symptoms (e.g. body image related symptoms and compensating behaviour) [15, 17]. Moreover, improvements in psychological well-being and quality of life were observed [15]. Of the 13 studies, five investigated adult inpatient samples. None of the studies investigated the course of illness following discharge from inpatient treatment. The authors of the reviews concluded that although initial studies showed promising results, more research is warranted to increase the understanding of compulsive exercise in eating disorders, associations with eating disorder pathology and influence on treatment outcome [15–17]. In order to contribute with increased knowledge to the field, the research focus in this article was directed towards negative and compulsive attitudes to exercise in female adult eating disorder inpatients through a naturalistic follow-up study.

The aims of the study were to:

1. Identify how various dimensions of attitudes towards compulsive exercise change between admission, discharge, and at follow-up in a transdiagnostic sample of hospitalized females with eating disorders.
2. Identify whether changes in attitudes to compulsive exercise predict the course of eating disorder psychopathology and BMI in relation to changes in general mental distress, interpersonal problems, and depression.

## Methods

### Participants and procedure

The study was conducted as part of a larger follow-up study, and participants in the cohort were recruited from an adult inpatient eating disorder unit in the Central Norway region. Between January 2005 and December 2013, a total of 128 patients were admitted to the unit. Six men were excluded because the EED questionnaire was only validated for women, and 6 female patients were excluded because they decided not to receive treatment after the introductory week. Of the remaining 116 female patients, 78 participated in the follow-up assessment, yielding a response rate of approximately 67 %. A subset of this sample (26 participants) was included in a former study investigating the impact of disturbed body image on treatment outcome [18]. No compensation was given for participation. Written invitations with study information, consent forms, and questionnaires were mailed to former patients for the follow-up assessment.

The data were collected at admission, discharge, and follow-up. The average follow-up period after discharge from treatment was 26 months (*SD* = 15 months, median = 22 months, range = 9.5–92.5 months). The main reason for the variety of the follow-up period is due to the study design. The follow-up study was approved in 2009. All former admitted patients were invited to participate, giving an extended follow-up period for the patients admitted in the earliest years. One of the participants had an extraordinarily long follow-up period of 92 months. The analysis of change during treatment and the analysis of predictors of outcome were performed both with and without the inclusion of the latter participant in the sample. The difference did not have a noteworthy effect on the results and data relating to the participant have been included in all reported results.

The baseline values for participants and non-participants were compared (t-test for independent groups). No differences were found between the groups: age (*t*(114) = 0.72, *p* = .472), body mass index (BMI) (*t*(114) = -1.3 7, *p* = .173), EED global score (*t*(114) = 0.65, *p* = .520) and Eating Disorder Inventory (EDI-2) (*t*(114) = 1.30, *p* = .197). Similarly, no differences between participants and non-participants were found in the discharge values: BMI (*t*(114) = -0.11, *p* = .910), EED global score (*t* (114) = 1.23, *p* = .221), and EDI-2 sum score (*t*(114) = 1.27, *p* = .205). Among the 78 participants, 78 % (*n* = 61) had completed treatment as planned, 17 % (*n* = 13) had discharged themselves from treatment, and 5 % (*n* = 4) had been discharged in accordance with routine procedures due to breaking the treatment contract.

Diagnostic assessment and clinical interviews at admission were performed by licensed psychologists or psychiatrists, in accordance with DSM-IV diagnostic criteria [1]. The diagnostic distribution in the sample was 59 % (*n* = 46) AN, and approximately 21 % (*n* = 16) in BN and EDNOS respectively. All patients were admitted to inpatient treatment due to having serious symptoms of ED and having received prior treatment without satisfactory effect. The mean duration of illness (self-reported) prior to admission was 5.3 years (*SD* = 3.8, range 1–20 years) and the mean duration of inpatient admission was 134 days (*SD* = 56, range 14–281 days). The mean admission time for AN participants was 149 days (*SD* = 61, range 14–281), BN 115 days (*SD* = 36, range 49–192), and EDNOS 111 days (*SD* = 48, range 15–184). Underweight participants were defined as having a BMI ≤18.5, which has been described by the World Health Organization (WHO) as the threshold for a healthy weight. In this sample, 72 % (*n* = 56) had a BMI below 18.5 at admission (*M* = 15.9, *SD* 1.6, range 12.4–18.5).

The study received approval from The Regional Committee for Medical and Health Research Ethics and all participants provided written informed consent. The baseline characteristics of the sample are presented in Table 1.

**Table 1:** Baseline values for the whole sample and diagnostic groups

	Alle patients	AN	BN	EDNOS			Effect size^3^
	*N* = 78	*n* = 46	*n* = 16	*n* = 16		Bonferroni Post hoc.	Eta squared adjusted for age
	Mean (SD)	Mean (SD)	Mean (SD)	Mean (SD)	*F*-value^1^ *p*-value^2^	*p*-value^2^	*η* ^2^
Age (years)	21.1 (3.9)	20.4 (3.3)	22.2 (4.5)	22.2 (4.8)	2.00		
Range	16.0 - 36.5	16.0 - 31.7	17.8 - 34.8	16.6 - 36.5			
BMI	17.7 (3.5)	15.5 (1.4)	20.7 (2.5)	20.8 (4.1)	46.10***	AN vs BN and EDNOS***	
Range	13.4 - 34.1	12.4 - 17.6	17.5 - 24.7	18.1 - 34.1			
EED global score	3.0 (0.8)	3.0 (0.7)	3.1 (1.0)	2.9 (0.9)	0.11		.00
EED compulsive	3.4 (1.2)	3.4 (1.2)	3.4 (1.2)	3.5 (1.1)	0.46		.00
EED positive	2.3 (1.4)	2.3 (1.2)	2.6 (0.9)	1.9 (0.9)	1.75		.04
EED bodily signals	2.5 (1.0)	2.6 (1.0)	2.3 (0.9)	2.5 (1.0)	0.32		.00
EED weight & shape	3.6 (1.2)	3.5 (1.2)	4.0 (1.1)	3.5 (1.0)	1.03		.04
EDI-2 sum score	111.2 (40.8)	105.5 (36.0)	138.8 (43.2)	100.1 (41.8)	5.23**	AN vs BN, and BN vs EDNOS*	.13
BAT sum score	66.0 (17.4)	63.8 (16.6)	72.3 (18.1)	66.2 (18.5)	1.44		.04
SCL-90 mean score	1.6 (0.7)	1.6 (0.6)	1.9 (0.7)	1.6 (0.8)	1.60		.04
BDI sum score	30.5 (10.4)	29.9 (8.2)	34.7 (13.1)	27.8 (12.5)	1.95		.05
IIP-64 mean score	1.6 (0.6)	1.6 (0.6)	1.8 (0.6)	1.6 (0.8)	0.79		.02

Note: ^1^One way ANOVA between diagnostic groups, df (2, 75). Bonferroni post hoc test, only significant relationships are reported

*AN* anorexia nervosa, *BN* bulimia nervosa, *EDNOS* eating disorder not otherwise specified

*BMI* Body mass index, *EED* Exercise and Eating Disorder, EDI-2- Eating Disorder Inventory, *BAT* Body Attitude Test

SCL-90: Symptom Checklist. *BDI* Beck Depression Inventory. IIP-64: Inventory of Interpersonal Problems

^2^ *p*-value: * *p* < .05, ** *p* < .01, and *** *p* < .001

^3^Effect size - Eta squared *η* ^2^: small effect size =: .02, medium = .13, and large = .26

### Treatment programme

The treatment programme was multidimensional and has been described in detail elsewhere [18]. In brief, the staff had multidisciplinary backgrounds and the treatment programme was based on psychodynamic theory, with elements of cognitive behavioural therapy and motivational interviewing. All participants were admitted voluntarily. Following an introductory week, each participant signed a contract formulating their individual goals and formally approving the terms of their treatment.

### Physical activity and exercise interventions

Physical activity and exercise interventions were an integrated part of the treatment programme from admission to discharge. According to procedure in the unit, the participants’ former exercise experiences were addressed individually at admission, including the reasons why they exercised, how their exercise affected other eating disorder symptoms and vice versa, and the amount of exercise they did. An individualized treatment plan and goals for physical activity as well as an exercise intervention were prepared for each participant.

Each week there was a structured programme with group therapy and individual therapy sessions, which also included body-oriented therapy, adjusted outdoor activity (e.g. walking, horse riding, climbing, kayaking, and skiing in the winter) and regular exercise groups. Underweight participants were required to have a minimum BMI of approximately 17.0 before participation in exercise groups and a planned weight gain had to be met in order to continue participation. Qualified personnel were responsible for planning and supervising the activities. The body-oriented therapy was based upon the theory and principles of Norwegian Psychomotor Physiotherapy (NPMP) [19], which in turn forms part of the Nordic physiotherapy and body-oriented therapy tradition [20]. The initial focus in body image therapy was upon relaxation and body awareness movements. All participants were gradually exposed to diverse approaches that address the relationships between the body, emotions, and interpersonal and social aspects. Regular exercise groups were offered later on in the treatment programme, with one hour of strength training and one hour of aerobic activity. These exercise groups were based on basic training principles such as muscular strength and endurance, cardiovascular endurance, variation, progression, and restitution, and were adjusted to individual needs and capacity. One of the aims was to expose the participants to different levels of intensity, but the exercise was mainly performed at a moderate level. The personnel facilitated a social arena in which participants could enjoy the activities and get new experiences in a non-competitive atmosphere. Individual goals and challenges were addressed before and after exercise sessions to help participants to integrate healthy attitudes and behaviour in accordance with their treatment plan. Participants also received individual supervision, during which a heart rate monitor could be used as an additional tool. Psychoeducation formed part of the intervention and included anatomy, physiology, what constitutes healthy exercise, and what is unhealthy exercise. At discharge, plans were made for the transference of the achieved changes to the participants’ daily life at home.

### Measures

#### Compulsive exercise

The Exercise and Eating Disorder (EED) self-report questionnaire [9] is a validated, short questionnaire. It has been developed to cover a broad perspective on attitudes towards compulsive exercise in eating disorders. It is the first clinically-derived questionnaire measuring attitudes towards compulsive exercise in patients with eating disorders and is intended for use in clinical settings [9]. It consists of 18 statements with a four-factor structure and a six-point response scale from zero to five (*never*, *rarely*, *sometimes*, *often*, *usually, and always*). The global score and subscale scores are based on mean values. Higher scores indicate greater compulsivity and unhealthy exercise. The subscales cover clinically relevant issues:

1. *Compulsive exercise* (being physically active to avoid dealing with negative emotions; if not active: it feels wrong, I don’t eat, I can’t relax, I get a bad conscience, my body feels big or nasty; and I listen to my body).
2. *Positive and healthy exercise* (enjoy being physically active; physically active to be healthy; like to exercise with other people).
3. *Awareness of bodily signals* (I notice when: I feel fit/am in shape, when I get tired, thirsty, or hungry).
4. *Exercise for weight and shape reasons* (active in order to: be thin, burn calories, and for appearance reasons).

The EED has been shown to have satisfactory psychometric properties. In the validation study, EED discriminated significantly (*p* < .001) between patients and controls on Global scale, subscales and all single items, showed good internal consistency (Chronbach’s alpha .90 in Global scale) and a satisfactory test-retest stability (Pearson’s correlation factor = .86 on Global score) [9]. The subscale on compulsive exercise showed the strongest correlation with eating disorder symptomatology (*r* (441) = 0.70, *p* < .01). In the current study, Cronbach’s alpha at admission was .86 for the EED global score, and subscale values were in the range of .65 to .90. At follow-up, the respective values were .94 (global score) and .75 to .94 (subscales). Three versions of the EED were used in the study. Despite some small changes to the wording across the revised versions, the meaning of the content has remained unchanged. Version 2 of the EED included three questions investigating the frequency, intensity, and duration of exercise, all of which have been validated and used elsewhere [21]. Due to differences in the way amount of exercise was reported in Versions 1 and 2 of the EED [9, 22], these quantitative measures are not presented in this article. During the study, the EED global score was used to predict BMI at outcome and follow-up, and the subscale on compulsive exercise was used as a predictor variable in the analyses using EDI-2 as the main outcome measure.

#### Disturbed body image

The Body Attitude Test (BAT) [23] is a short self-report questionnaire addressing subjective body experience and the attitude that a person with eating disorders has towards their body. It is a self-report measure with 20 items measured on a six-point response scale from *never* to *always.* The items are divided into four subscales: (1) negative appreciation of body size, (2) lack of familiarity with one’s own body, (3) general dissatisfaction, and (4) a rest factor with two items which is not considered a separate subscale. The BAT has shown good validity and reliability [23, 24]. Cronbach’s alpha values at admission and follow-up for BAT sum score were .92 and .96, while the respective values for the *lack of familiarity with one’s own body* subscale were .82 and .92. The BAT has been found to be a significant predictor of changes in EDI-2 from admission to discharge, and most of the predictive value was represented by the BAT subscale on lack of familiarity with one’s own body [18]. Therefore, the BAT sum score and *lack of familiarity with one’s own body* subscale were respectively selected as covariates for the outcome variables BMI and EDI-2.

#### Eating disorder symptoms

The Eating Disorder Inventory (EDI-2) [25] is a widely used self-report tool with 91 items and 11 subscales, covering eating disorder symptoms, attitudes, and behaviours. EDI-2 does not cover exercise-related issues. EDI-2 has been validated in Nordic samples [26, 27]. Cronbach’s alpha values at admission and follow-up were .95 and .98. The EDI-2 sum scores were one of the outcome measures, and EDI-2 was included as covariate in the analyses with BMI as outcome measure.

#### General psychopathology

The Symptom Checklist 90 revised version (SCL-90R) [28] was used to measure general psychopathology. The SCL-90R is a validated self-report tool with 90 items divided into 11 subscales [29]. In the study, we only report the mean score of the severity index. Cronbach’s alpha values at admission and follow-up were .97 and .99. The mean scores of the SCL-90R were included as a covariate in both models.

#### Depression

The Beck Depression Inventory (BDI) [30] is a validated self-report inventory with 21 questions measuring the severity of depression. The BDI global score was included as a covariate in both models. Cronbach’s alpha value at admission was .89.

#### Interpersonal problems

The Inventory of Interpersonal Problems (IIP-64) [31] was used as a measure of interpersonal problems. The IIP-64 is a validated self-report inventory consisting of 64 items and eight subscales. Cronbach’s alpha values were .96 at admission and follow-up. The mean scores of the IIP-64 were included as covariates.

#### Body mass index

BMI is calculated using the following formula: kg/m^2^. A BMI of 20.0 was the target BMI in the inpatient unit. During treatment, participants were weighed in the morning before breakfast, while wearing only undergarments. At follow-up, body weight was self-reported. When using BMI as the outcome measure, analyses were performed for all underweight patients (admission BMI ≤18.5). The BMI for the whole sample was included as a covariate in the analyses with EDI-2 as an outcome measure. Weight gain during treatment for eating disorders is especially associated with AN. To provide additional information, separate analyses including only AN participants were performed.

### Statistical analysis

The Kolmogorov-Smirnov test confirmed normality for the EED global scores at admission, discharge, and follow-up (*D*(77) = .066 – .080, *ps* = .200). The following analyses were performed: descriptive analyses, one-way ANOVA with the Bonferroni post-hoc test, paired sample *t*-test, and Cronbach’s alpha. Effect sizes were calculated using Cohen’s *d* [32]. Linear mixed model regression analyses were used to investigate the predictive value of the EED. Due to the small size of the diagnostic groups and the lack of significant differences in the EED global scores between groups at admission (*p* = .89), these analyses were performed for the whole sample. In the linear mixed models regression analyses, three models were explored and the results reported: (1) the null model represented the intercept values of the outcome measure and its variances, (2) Model 1 included the covariate of particular interest (i.e. the EED score), and (3) in Model 2 values were adjusted for all significant covariates. R-squared was calculated based on differences of variance between the null model and the other models, and R-squared, the likelihood ratio test (LR test), change in variances, and log-likelihood values were used as measures of model improvement. The intraclass correlation (ICC), which represents the within-cluster correlation, is reported. Significance levels were set at *p* < .05, and data were analysed and presented for the whole sample and subgroups using SPSS Version 21 and Stata/MP 13 software.

### Missing data

In total, the amount of missing data in the returned self-report questionnaires was less than 5 %. Missing data were handled in two ways. Missing single items were replaced by a calculated mean for the specific subscales for each participant. Admission data were completely missing for two participants, while for one participant the discharge data were missing. Data at one or two measure points were missing for eight other participants. Evaluation of the data showed that the missing data were missing at random, and therefore multiple imputations could be performed to replace them.

## Results

### Study aim 1 – change of measures

The mean values of the EED, global score, and subscales improved significantly (*ps* < .01 to *ps* < .001) from admission to discharge and admission to follow-up. The effect size was large for the global score (.93) and medium to large for the subscales (.49–.84). From discharge to follow-up, the changes were smaller, yet significant for the EED subscales: *compulsive exercise, awareness of bodily signals*, and *exercise for weight and shape reasons* (*ps* < .05). The effect sizes were small (.26–.32).

Significant changes (*p* < .001) were observed for the measures of general psychopathology, body image, and eating pathology across the three time points. Effect sizes from admission to follow-up were large for the BDI (1.00), medium to large for EDI-2, the BAT sum score, the *lack of familiarity with one’s own body* subscale, and the SCL-90R (.74, .66, .73, and .63, respectively), and between low to medium for the IIP-64 (0.43). From discharge to follow-up, there were significant changes only in the BAT sum score, with small effect size (.29). No significant changes were observed for the EDI-2, BDI, SCL-90R, and IIP-64 during the follow-up period. The scores at admission, discharge, and follow-up are summarized in Table 2.

**Table 2:** EED scores (global score and subscales) for the whole sample at admission, discharge and follow-up and differences from admission to follow-up

	Admission *N* = 78		Discharge *N* = 78		Follow-up *N* = 78		Diff^1^ A - F	Effect size A - F Cohen’s *d* ^3^
	Mean (SD)	95 % CI	Mean (SD)	95 % CI	Mean (SD)	95 % CI	*t* *p*-value^2^	
EED Global score	3.03 (0.81)	2.83; 3.21	2.34 (0.86)	2.15; 2.54	2.13 (1.08)	1.89; 2.37	6.90***	.93
EED Compulsive	3.43 (1.18)	3.16; 3.70	2.68 (1.28)	2.40; 2.98	2.33 (1.43)	2.03; 2.67	6.80***	.84
EED Positive and healthy	2.31 (1.12)	2.05; 2.56	1.55 (0.93)	1.33;1.74	1.77 (1.07)	1.52; 2.00	3.58**	.49
EED Bodily signals	2.50 (0.98)	2.27; 2.71	1.96 (0.89)	1.75; 2.15	1.63 (1.13)	1.35, 1.86	6.17***	.82
EED Weight and shape	3.53 (1.24)	3.30; 3.86	2.94 (1.26)	2.64; 3.21	2.59 (1.47)	2.27; 2.93	5.94***	.69

^1^Paired sample t-test from admission to follow-up A: Admission, F: Follow-up

^2^ *P*-values: * *p* < .05, ** *p* < .01, and *** *p* < .001. ^3^Cohens *d*: Small effect size = .20, medium = .50, and large = .80

### Study aim 2

#### EDI-2 as outcome measure

The initial analyses indicated multicollinearity between the EED global score and BAT sum score. These global scores were therefore replaced with the EED subscale on compulsive exercise and the BAT subscale on lack of familiarity with one’s own body as covariates. In the null model, EDI-2 scores from admission to follow-up improved significantly (*p* < .001), but with large values of unexplained variance. Model 1 showed model improvement, R-squared = 47.5 %, and the EED compulsive exercise was a significant predictor (*z* = 15.22, *p* < .001). In Model 2, all covariates except BMI were found to contribute significantly (*p* < .001) and R-squared = 82.3 %. In this model, the EED compulsive exercise score remained significant, but with lower values (z = 2.92, *p* < .01). The reduced z-value indicated shared content between EED with the other covariates, but also accounted for unique explained variance. Significant LR test (*p* < .001) as well as reduced variance and log-likelihood values, and increased R-squared all indicated a further improvement of model fit to the data. The addition of a patient-specific random slope indicated further model improvement (LR test, *p* < .01). The results for the models are presented in Table 3.

**Table 3:** Random intercept models with EDI-2 as outcome measure for the whole sample

Fixed part	Null model			Model 1			Model 2		
	Coefficient (SE)	*z*-value *p*-value^3^	95 % CI	Coefficient (SE)	*z*-value *p*-value^3^	95 % CI	Coefficient (SE)	*z*-value *p*-value^3^	95 % CI
Intercept	90.66 (4.25)	21.32***	82.33; 98.99	18.56 (5.69)	3.26**	7.38; 29.72	-3.95 (4.08)	-0.97	11.95; 4.03
EED compulsive				25.55 (1.68)	15.22***	22.25; 28.83	4.58 (1.57)	2.92**	1.50; 7.66
BAT familiarity							1.18 (0.30)	3.93***	0.59; 1.77
BDI sum score							1.09 (0.19)	5.75***	0.72; 1.47
SCL-90R mean score							12.15 (4.05)	3.00**	4.20; 20.10
IIP-64 mean score							13.23 (3.81)	3.48**	5.77; 20.70
Random part									
^ 1^ψ variance	927.42			548.36			126.65		
^ 2^θ variance	1447.78			695.34			294.33		
Derived estimates									
R-squared				47.5			82.28		
ICC	0.39						0.30		
Log likelihood	-1225.35			-1145.06			-1029.47		

*EED* Exercise and Eating Disorders, *BAT* Body Attitude Test, *BDI* Beck depression Inventory, SCL-90R: Symptom checklist revised, IIP-64: Inventory of interpersonal problems

^1^ψ Variance: Variance between patients. ^2^θ Variance: Variance within observations in patients

*ICC* Intraclass correlation. ^3^ *P* values: * *p* < .05, ** *p* < .01, and *** *p* < .001

#### BMI as outcome measure (admission BMI (<18.5, *n* = 56))

No signs of multicollinearity were present in these analyses, and the EED global score and BAT sum score were used as covariates in the models. Model 1 showed improved model fit to the data and R-squared = 24.2 %. The EED global score was a significant predictor of change in BMI (*z* = -7.29, *p* < .001). The negative z-value indicated an association between reduced EED global score and increased BMI. In Model 2, non-significant covariates (EDI-2, SCL-90, and IIP-64) were removed. Each of these variables was separately included in the model once more, without any change in significance level. Additional significant covariates retained in Model 2 included BAT sum score (*p* < .001) and BDI (*p* < .01), and R-squared = 36.5 %. The EED global score continued to be a significant predictor in Model 2 (*z* = -5.78, *p* < .001). Adjusted for the other covariates, the z-value of the EED was attenuated by 1.51, indicating some shared content of the measures, but also confirmed the importance of the EED as a unique and significant predictor of improvement in BMI. Reduced variance and log-likelihood, increased R-squared, and significant LR test (*p* < .001) all confirmed improvement in the model fit to the data. Inclusion of a patient specific slope did not improve any of the models significantly. Additional analyses of the AN group showed almost similar values to those in the whole underweight group. The results of the models for the whole underweight group and the AN group are presented in Table 4. The values confirmed that Model 2 provides the best fit to the data.

**Table 4:** Random intercept models, BMI as outcome measure for patients with BMI ≤ 18.5 at admission (*n* = 56) and AN patients (*n* = 46)

Fixed part	Null model			Model 1			Model 2		
	**Null model** **AN** ^**a**^			**Model 1 AN**			**Model 2 AN**		
Intercept	Coefficient (SE)	*z*-value *p*-value^3^	95 % CI	Coefficient (SE)	*z*-value *p*-value^3^	95 % CI	Coefficient (SE)	*z*-value *p*-value^3^	95 % CI
BMI	18.53 (0.28)	67.86***	19.04; 20.18	21.80 (0.49)	44.92***	20.86; 22.76	20.66 (0.52)	33.79***	19.64; 21.68
	**18.33 (0.25)**	72.63***	**17.84; 18.83**	**21.83 (0.59)**	36.72***	**20.67; 23.00**	**20.68 (0.61)**	33.90***	**19.48; 21.87**
EED global score				-1.37 (0.19)	-7.29***	-1.73, -1.00	-1.67 (0.29)	-5.78***	-2.24; -1.10
				**-1.45 (0.23)**	-6.34***	**-1.90; -1.00**	**-1.82 (0.34)**	-5.34***	**-2.49; -1.15**
BAT sum score							0.07 (0.01)	4.85***	0.04; 0.10
							**0.08 (0.02)**	4.85***	**0.05; 0.11**
BDI sum score							-0.10 (0.02)	-4.96***	-0.14; -0.06
							**-0.11(0.24)**	-4.60***	**-0.16; -0.06**
Random part									
^ 1^ψ variance	0.00				0.00		0.27		
	**0.00**				**0.00**		**0.31**		
^ 2^θ variance	7.49				5.68		4.49		
	**8.34**				**6.39**		**4.88**		
Derived estimates									
R-squared					24.2		36.5		
					**23.5**		**37.8**		
ICC	0.00				0.00		0.06		
	**0.00**				**0.00**		**0.06**		
Log likelihood	-405.1				-382.0		-366.9		
	**-324.9**				**-307.4**		**-293.7**		

^a^All values of AN patients are written in bold

*EED*, Exercise and Eating Disorders, *BAT* Body Attitude Test, *BDI*: Beck depression Inventory

^1^ψ Variance: Variance between patients. ^2^θ Variance: Variance within observations in patients

*ICC* Intraclass correlation. ^3^ *p*-values: * *p* < .05, ** *p* < .01, and *** *p* < .001

## Discussion

Our results showed significant improvement across different dimensions of attitudes towards compulsive exercise in the patient cohort during inpatient treatment and follow-up. Overall, significant changes were either maintained or continued to show additional improvement at follow-up. Further, the results demonstrated that improvements in these compulsive exercise dimensions significantly predicted the course of the illness, as measured by the EDI-2 and BMI. Regarding BMI as outcome measure, additional analyses of the AN group showed almost similar results to those for the whole underweight group.

Although different measures were used, the positive change found in attitudes towards compulsive exercise during treatment is consistent with earlier research [8, 14]. Our follow-up data are the first evidence of show how such attitudes develop in the course following discharge from inpatient treatment. The improvements were significant in three out of four subscales of EED and in the BAT sum score, compared to no changes in other measurements.

Meyer and colleagues [4] propose that weight and shape issues, regulation of negative affect and rigidity, and perceived negative consequences when exercise is restricted or stopped are factors that should be investigated in research directed towards exercise in eating disorders. These factors are in accordance with the view that qualitative measures of excessive exercise are clinically most important [3]. Our research has been conducted in accordance with these recommendations and perspective. The lack of other long-term follow-up studies investigating attitudes towards compulsive exercise limited our ability to compare our findings to existing research directly. However, our results indicate that changes in attitudes towards compulsive exercise during treatment and follow-up may prove to be a beneficial factor that would facilitate treatment progress and outcome. Similar trends have been found in other studies that have reported results during treatment [15–17]. Two studies with comparable treatment settings were found [8, 14]. Despite differences in sample sizes and sample characteristics, as well as some inconsistencies in definitions and measurements, these studies found a significant association between reduced exercise dependency and reduced eating disorder symptoms during treatment. The longitudinal design of our study has built upon and extended earlier research, and the main findings emphasize the importance of attitudes towards compulsive exercise for the outcome of eating disorder symptoms following discharge.

To date, researchers have directed more attention towards the relationship between weight gain and exercise interventions in treatment programmes for patients with AN. The results of their research have indicated that the incorporation of exercise interventions in treatment does not have an adverse effect on achieving weight gain [15–17]. Our findings support the existing literature, and the follow-up data strengthen and extend the knowledge in the field.

Relapse after inpatient treatment is common and it has been suggested that it is important to take exercise-related elements into consideration [11, 12]. Moola and colleagues [15] have stated that if patients’ exercise beliefs are allowed to remain unchanged during inpatient treatment that might increase their risk of continued unhealthy exercise after discharge. The extent to which improvements in negative attitudes towards compulsive exercise persist following discharge has not been reported previously, nor have earlier studies investigated the predictive significance of attitudes towards compulsive exercise on eating pathology following treatment.

Compulsive exercise is a complex phenomenon. In our study, different domains of attitudes towards exercise were shown to be significant predictors of changes in eating pathology and BMI. In the first model, using the EDI-2, the compulsive exercise subscale of the EED was used as covariate and the results indicated a significant association between compulsive elements of exercise and eating disorder symptoms. In the second model, using BMI as the outcome variable, the EED global score significantly predicted weight gain, thus highlighting the relationship between BMI and various dimensions of attitudes towards exercise. It is important to note that these values were almost similar in the whole underweight group and in additional analyses which included only AN participants. The proportion of explained variance increased in both models when other measures were added as covariates, from 47.5 to 82.3 % (EDI-2 as outcome measure) and 24.2 to 36.5 % (BMI as outcome measure). These findings illustrate the importance of the other covariates, yet exercise remained a significant predictor, indicating that addressing compulsive exercise and attitudes towards exercise in the treatment of eating disorders may be a useful intervention.

### Strengths and limitations

The study was a naturalistic cohort study investigating a medium-size sample of female inpatients with eating disorders. All admitted patients were invited to participate. None of the participants were excluded due to symptoms or comorbidity, and this has probably improved the representativeness of our sample and generalizability of our findings to other inpatient settings. The inclusion of follow-up data is a notable strength and adds significantly to existing knowledge of longer term outcomes and compulsive exercise.

However, it is necessary to consider several limitations of the study. The study was a cohort study with no control group and it is uncertain whether the findings related specifically to our treatment programme, which included a physical activity component, or whether they indicate a general pattern of associations between various aspects of eating disorders over time. All participants and only females were recruited from the same inpatient unit, and the findings may not be generalizable to outpatient or other treatment settings. The ability to generalize is also limited by the fact that the EED has only been validated in women. The follow-up of 67.2 % is a weakness, yet no baseline differences were found for age, BMI, EED sum global scores, and EDI-2 scores, or for discharge scores for EED and EDI-2 between participants compared to non-participants. Great variety in lengths of follow-up may also affect the results. Trained and specialized clinicians provided diagnostic quality, yet the diagnostic procedures did not include structured interviews. A transdiagnostic sample was used for the majority of the analyses in the study, due to a lack of diagnostic group differences in EED scores at baseline. Our results might have been influenced by the small sample size of BN and EDNOS participants.

The quantity dimension of exercise was not reported in the study, which may be considered a limitation. Due to differences in how this behaviour has been reported in EED Versions 1 and 2, we decided not to include this information. However, in the validation study an association was found between EED scores and high-level exercise (frequency, intensity, and duration). The majority of patients who were high-level exercisers had high EED scores, whereas among controls the majority of such high-level exercisers had low EED scores. From a clinical perspective, defining the level of exercise as harmful or not should also be evaluated in relation to somatic conditions (e.g. degree of underweight), fitness level (e.g. athletes), and former exercise behaviour [9].

Admission time showed great variation in the study, and prolonged admissions were mainly due to the time necessary to restore weight in severely underweight AN patients, but also related to symptoms of comorbidity. The quiet long admission time may limit the generalizability of the results compared to other shorter inpatient treatment program. There was only one measurement following discharge and the duration of the follow-up interval varied across individual participants. It is important for our results to be replicated in a sample that is large enough to examine different diagnostic groups and different follow-up periods.

So far, there have been no published reports of randomized control trials in which exercise intervention in an inpatient treatment programme has been compared to no exercise intervention, and this makes it difficult to draw conclusions about the impact of exercise interventions. However, the findings in our study show the importance of attitudes towards compulsive exercise for outcome in participants with eating disorders and support the relevance of assessing and addressing attitudes towards compulsive exercise during treatment.

## Conclusions

We found a significant reduction in thoughts and attitudes towards compulsive exercise during treatment and that these changes predict outcome, as indicated by reduced eating disorder pathology and increased BMI for underweight participants. The results of the study have implications for future research. In order to evaluate the impact of exercise interventions on outcome, it is important to investigate gender differences, to include a control group and to investigate the relationship to both quality and quantity dimensions.

## Abbreviations

AN: anorexia nervosa
BAT: Body Attitude Test
BDI: Beck Depression Inventory
BMI: body mass index
BN: bulimia nervosa
DSM: diagnostic and statistical manual of mental disorders
EDI: Eating Disorder Inventory
EDNOS: eating disorder not otherwise specified
EED: Exercise and Eating Disorder
ICC: intraclass correlation
IIP: Inventory of Interpersonal Problems
LR: likelihood ratio
NPMP: Norwegian Psychomotor Physiotherapy
SCL: Symptom Checklist
